# Resveratrol enhanced mitochondrial recovery from cryopreservation‐induced damages in oocytes and embryos

**DOI:** 10.1002/rmb2.12401

**Published:** 2021-06-27

**Authors:** Hisataka Iwata

**Affiliations:** ^1^ Tokyo University of Agriculture Atsugi City Japan

**Keywords:** cryopreservation, mitochondria, resveratrol, SIRT1

## Abstract

**Background:**

Mitochondria play a crucial role in nuclear maturation, fertilization, and subsequent embryo development. Cryopreservation is an important assisted reproductive technology that is used worldwide for humans and domestic animals. Although mitochondrial quantity and quality are decisive factors for successful development of oocytes and embryos, cryopreservation induces mitochondrial dysfunction. Upon thawing, the damaged mitochondria are removed, and de novo synthesis occurs to restore the function of mitochondria. Resveratrol, 3,5,4′‐trihydroxystilbene, is a polyphenolic antioxidant that has versatile target proteins, among which sirtuin‐1 (SIRT1) is a key regulator of in mitochondrial biogenesis and degradation.

**Methods:**

The present study is a literature review focusing on experiments involving the hypothesis that the activation of mitochondrial biogenesis and degradation following cryopreservation and warming by resveratrol may help mitochondrial recovery and improve oocyte and embryo development.

**Main findings and conclusion:**

Resveratrol improves oocyte maturation and development and upregulates mitochondrial biogenesis and degradation. When vitrified‐warmed embryos are treated with resveratrol, it helps in mitochondrial regulation and recovery of embryos from cryopreservation‐induced damage.

**Conclusion:**

Resveratrol treatment is a possible countermeasure against cryopreservation‐induced mitochondrial damage.

## Graphical abstract

Cryopreservation‐induced damage in embryos may be ameliorated by an activation of mitochondrial synthesis and degeneration.

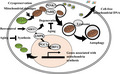

## 1 INTRODUCTION

Embryo transfer (ET) is a major assisted reproductive technology (ART) used for domestic animals and humans worldwide. In industrialized countries, an increasing number of women get pregnant using ET technology. Success in ET depends on the quality of embryos, which are affected by physiological conditions of oocyte donors such as obesity and diabetes, maternal age, and an ART per se, including in vitro culture and cryopreservation. Cryopreservation is pivotal in ART and supports the widespread use of the embryos collected from genetically and/or commercially valuable domestic animals, as it increases opportunity for pregnancy. Cryopreservation methods have improved over decades; oocytes that are usually difficult to cryopreserve now have high survival rate (over 80%) after vitrification,^1^ and no differences have been observed in the clinical live birth rate between fresh and vitrified blastocyst transfer case.^2^ However, in the clinical ET, the background of recipient women such as selection of cycle and conditions differs between fresh and vitrified ET. In a study on Nelore cows, the pregnancy rate of vitrified embryos was found to be 5% lower than that of fresh embryos.^3^ In line with these results, a national survey of bovine ET outcomes in Japan revealed that the pregnancy rate of frozen ET was 5% lower than that of fresh counterparts (http://www.maff.go.jp/j/chikusan/sinko/lin/l_katiku/attach/pdf/index‐10.pdf). Therefore, it is important to address that cryopreservation‐induced damage in embryo and oocytes and develop possible countermeasures. Of the different types of damages detected in cryopreserved‐warmed oocytes and embryos, mitochondrial dysfunction and high reactive oxygen species (ROS) are key concerns. A study in humans showed that vitrified‐warmed oocytes have low ATP levels and mitochondrial membrane potential, abnormal mitochondrial configuration and localization, and ROS and intracytoplasmic Ca levels.^4^, ^5^, ^6^ In addition, transition electron microscope revealed that mitochondria in vitrified‐warmed embryos had swollen morphology.^7^, ^8^, ^9^ In our studies based on porcine oocytes, bovine eight‐cell stage and blastocyst stage embryos, ATP content, mitochondrial genome integrity and developmental ability in cryopreserved samples was low compared with those fresh counterparts.^10^, ^11^, ^12^ A previous study showed that vitrification of ovine oocytes induced low mitochondrial function and high ROS content; however, optimal restoration period, in this case 4 hours after vitrification‐warming partly improved oocyte developmental ability.^13^ However, little is known about the restoration of mitochondria after the warming of oocytes and embryos. Based on evidences supporting that all frozen‐warmed embryos successfully give rise to a healthy offspring, it is highly plausible that oocytes and embryo undergo cryopreservation‐induced damage for a certain period; however, damaged related alterations can be overcome during subsequent embryo development. Therefore, reduction in harmful factors associated from vitrification or augmentation of mitochondrial recovery from vitrification‐induced damage are the possible countermeasures to achieve high pregnancy rates and produces healthy offspring. Several studies have been conducted to address this issue, which include investigation of natural compounds as antioxidants and their ability to activate mitochondrial biogenesis and degradation. Here, this review highlights a possible strategy to combat vitrification‐induced injuries, particularly focusing on resveratrol (3,5,4′‐trihydroxystilbene) that can reduce reactive oxygen stress and activate mitochondrial biosynthesis and degradation.

## 2 RESVERATROL AND POSSIBLE TARGETS

Resveratrol is a polyphenolic antioxidant found in diverse plants.^14^ Plants synthesize resveratrol through the enzyme stilbene synthase in response to several stresses conditions such as ultraviolet irradiation, temperature, mechanical damage, and interaction with microorganisms.^15^ Resveratrol has been widely studied in the context of cardiovascular diseases,^16^ metabolic syndrome and diabetes, neurogenerative and inflammatory diseases,^17^ cancer, and age‐related deterioration.^18^, ^19^ Moreover, it is possible to identify the resveratrol‐interacting proteins using the DAVIDs genetic association data base^20^, ^21^ and the InterPro data base.^22^ Further, the proteins targeted by resveratrol have been annotated to disease categories such as cancer, immune‐related disorders, neurological disorders, aging, and infection. Additionally, these have been classified into protein superfamilies; zinc finger, nuclear hormone receptor, p53, EGF receptor, IGFBP, cytochrome p450, NF kappa β, sirtuin, nitric oxidase,^23^ indicating the versatile functions of resveratrol. Of these proteins, sirtuin is a well‐known target of resveratrol. The sirtuin family plays an important role as caloric restriction mimetics. It comprises a group of highly conserved class 3 histone deacetylases consisting of seven members, SIRT1‐SIRT7. Resveratrol can directly activate SIRT1 and 5. SIRT1 exhibits a lifespan‐extending effect in mice.^24^ It deacetylates proliferator‐activated receptor‐γ co‐activator 1α (PGC1a), a master regulator of energy metabolism, and increases mitochondrial function and biogenesis by activating NRF1, NRF2, and downstream genes such as *TFAM*.^23^, ^25^ SIRT1 KO mice have exhibited reduced mitochondrial number,^26^ that is, resveratrol cannot induce mitochondrial synthesis in such mice.^25^ Therefore, resveratrol is considered not only as an antioxidant but also as a chemical activator for mitochondrial biogenesis.

## 3 ANTIOXIDANTS PROTECT VITRIFIED‐WARMED OOCYTES AND EMBRYOS

There is substantial literature that suggests that vitrification of oocytes induces ROS generation and subsequently reduces oocyte quality and subsequent development.^27^, ^28^ Therefore, the effect of antioxidants on oocytes and embryo following vitrification has been extensively studied; supplementation of culture medium of vitrified‐warmed oocytes or embryos with melatonin, glutathione ethyl‐ester, N‐acetyl cysteine, α‐tocopherol, or coenzyme Q reduced ROS levels and improved oocyte viability as well as embryo development in equine, cows, and mice.^6^, ^29^, ^30^, ^31^, ^32^ In addition, supplementation of vitrified‐warming solution with acetyl‐L‐carnitine, N‐acetyl‐cysteine, and lipoic acid improved the cell number of the blastocysts and fetal development in mice.^33^ Furthermore, addition of vitamin E to the culture medium of vitrified‐warming ovarian tissue improved oocyte and subsequent blastocyst stage development.^34^ In line with these trials, resveratrol has been used as an antioxidant to reduce ROS levels. Supplementation of in vitro maturation medium of porcine oocytes with resveratrol reduced ROS levels and increased glutathione (GSH) content in oocytes and improved subsequent embryonic development of in vitro‐fertilized or parthenogenetically activated porcine embryos.^35^, ^36^ Likewise, supplementation of maturation medium of porcine oocytes with resveratrol reduced ROS content and increased expression levels of genes associated with antioxidants; this improved the subsequent development of somatic nuclear‐transferred embryos.^37^ In addition, resveratrol decreased ROS levels and increased GSH and *SIRT1* expression levels in bovine oocytes.^38^ Furthermore, culturing bovine embryos with resveratrol improved the developmental rate to the blastocyst stage by reducing ROS content and increasing ATP generation; the effect of resveratrol was diminished by SIRT1 inhibitor treatment.^39^ In similar context, incubation of vitrified‐warmed oocytes with resveratrol reduced ROS content and oxidative marker (γH2A) levels; however, GSH content increased and the subsequent embryo development improved in mice and pigs.^27^, ^40^, ^41^ In addition, blastocysts developed with resveratrol exhibited high cryo‐tolerance with improved subsequent developmental ability; moreover, ameliorated vitrification‐induced mitochondrial dysfunction and abnormal gene expression were observed.^42^, ^43^, ^44^ However, the molecular mechanism underlying the reduction of reactive oxygen levels upon resveratrol treatment is still unclear. Since the antioxidant capacity of resveratrol is far lower (16 times) than that of α‐tocopherol,^45^ other molecular mechanisms should be investigated.

## 4 MITOCHONDRIAL DYSFUNCTION AND DEGRADATION

Mitochondria are metabolic hubs play various roles in ATP production, lipid metabolism, Ca regulation apoptosis, and autophagy.^46^ A high number of mitochondria in oocytes indicates their good quality.^47^, ^48^, ^49^ Mitochondrial number and quality are strictly regulated; upon being damaged, these are removed from the cells through mitophagy. Mitochondrial membrane potential (MMP) is crucial for ATP generation, and loss of the MMP is the starting point of mitochondrial removal.^50^ Treatment with carbonyl cyanide m‐chlorophenyl hydrazine (mitochondrial uncoupler, CCCP) induces recruitment of parkin E3 ubiquitin ligase and activates proteasomal degeneration of the outer mitochondrial membrane, which is an initial stage of mitochondrial removal through mitophagy.^51^ Therefore, CCCP treatment is a mimetic of mitochondrial dysfunction and is used to study mitochondrial quality control systems.^52^ Likewise, the treatment of porcine cumulus cell oocyte complexes (COCs) with CCCP reduced mitochondrial ATP generation and increased both mitochondrial biogenesis and degradation in the oocytes. During this process, expression levels of genes associated with mitochondrial biogenesis and phosphorylated AMPK and SIRT1 are upregulated.^53^ Mitochondrial number is maintained through biphasic pathways (de novo synthesis and degradation), and measurement of mitochondrial DNA copy number is insufficient to estimate activity of synthesis and degradation in oocytes. Sato et al^54^ revealed that incubation of porcine oocytes with the proteasome inhibitor, MG132, inhibited mitochondrial degradation; hence, the total mitochondrial DNA copy number increased due to mitochondrial synthesis. In this context, they cultured porcine oocytes in a medium containing a selective combination of MG132 (an inhibitor of proteasome) and resveratrol (SIRT1 activator) and EX527 (SIRT1 inhibitor) and reported that upregulation of SIRT1 upon resveratrol treatment increased both mitochondrial biogenesis and degeneration. Furthermore, the expression levels of SIRT1 in oocytes were positively correlated with the mitochondrial copy number. Macroautophagy of mitochondria, known as mitophagy, plays a major role in mitochondrial degradation.^55^ Additionally, it has been reported that resveratrol induces autophagy and protects H9C2 cardiac myoblast cells from ischemic stress.^56^ Further, resveratrol activates autophagy through the AMPK/SIRT1 pathway, which plays a pivotal role in neuroprotection.^57^ Zhou et al showed that resveratrol treatment increased LC3‐2/LC3‐1, PINK 1, and autophagosome expression levels, and suggested that the activation of autophagy by resveratrol treatment protected oocytes from in vitro aging‐associated defects, including abnormal spindle formation, unstable cortical granule distribution, and decreased in ATP and mitochondrial DNA copy number.^58^ In addition to the substantial literature on mitochondrial degradation, monitoring the mitochondrial kinetics in oocytes and embryos requires complex evaluation processes; however, the lack of noninvasive markers hampers the study of mitochondrial degradation in oocytes and embryos following vitrification and warming. Mitochondria‐derived DNA is found in circulation^59^ and is detected in follicular fluid^60^ and in medium of human embryos and porcine granulosa cells.^61^, ^62^, ^63^ The amount of mitochondrial cell‐free DNA in the medium can be easily measured, and it is a possible useful marker to gain insight into mitochondrial kinetics, particularly, after mitochondrial damage. Interestingly, when cumulus oocyte complexes were treated with a mitochondrial uncoupler (CCCP) to induce mitochondrial dysfunction, the amount of mitochondrial cell‐free DNA increased in the medium.^61^ Furthermore, after induced mitochondrial dysfunction of granulosa cells, the excretion of mitochondrial cell‐free DNA into the culture medium increased in response to inhibition of autophagy (bafilomycin treatment), and significantly decreased in response to either MG132 (a proteasome inhibitor) or GW4869 (an inhibitor of intracellular vesicle formation).^62^ These results suggest that the amount of cell‐free mitochondrial DNA in the culture medium reflects the mitochondrial quality control processes of cells. Recently, Choong et al reported that CH12 cells secrete mitochondria into the culture medium, and the extent of the mitochondrial secretion increased upon treatment of the cells with CCCP or by suppression of PRKN and BNIP3 expression; however, the secretion was decreased by overexpression of PRKN and BNIP3, and it was concluded that the release of mitochondria into the culture medium is a distinct mitochondrial quality control pathway.^64^ In this context, resveratrol increased mitochondrial cell‐free DNA in the culture medium of cryopreserved‐warmed embryos and concomitantly, decreased mitochondrial DNA copy number and protein levels in the embryos.^10^, ^11^

## 5 RESVERATROL‐INDUCED ACTIVATION OF MITOCHONDRIAL BIOGENESIS AND DEGRADATION IN VITRIFIED‐WARMED OOCYTES AND EMBRYOS

It has been demonstrated that resveratrol improves embryonic development and enhances mitochondrial biogenesis and degradation in fresh oocytes.^54^ Likewise, Ito et al found that resveratrol treatment of vitrified‐warmed porcine oocytes activated genes related to mitochondrial synthesis, increased mitochondrial protein content and DNA copy number, and improved survival of the oocytes.^12^ Vitrification of bovine 8‐cell stage embryos induced mitochondrial dysfunction with low ATP levels and mitochondrial DNA integrity, which further resulted in a low developmental rate to the blastocyst stage. Incubation of these vitrified‐warmed embryos with resveratrol improved embryo development and, interestingly, the resultant blastocysts had fewer mitochondrial DNA copy number per blastomere; a high amount of mitochondrial cell‐free DNA was observed in the corresponding culture medium. These findings indicate that resveratrol induces the removal of vitrification‐induced damaged mitochondria from embryos and helps in their recuperation.^10^ Consistent with these findings, incubation of frozen (slow cooling) thawed bovine blastocysts in culture medium containing resveratrol improved the survival rate and subsequent development; however, the blastocysts had reduced mitochondrial number, and the corresponding culture medium had increased cell‐free mitochondrial DNA copy number in comparison with that observed for the culture without resveratrol treatment.^11^ During the clinical use of ET, embryos are transferred immediately after thawing, and long in vitro incubation of post‐thawed embryos is avoided in both cows and humans. Therefore, treatment of embryos with resveratrol before cryopreservation is more acceptable. In this context, bovine blastocysts were preincubated with resveratrol for 6 or 24 hours before slow freezing. Even a short incubation with resveratrol upregulated the expression levels of SIRT1, increased mitochondrial synthesis in embryos, and improved the survival rate of the frozen thawed embryos and pregnancy outcome following ET.^65^ In addition, pretreatment of bovine 8‐cell embryos with resveratrol before vitrification induced mitochondrial degradation and biogenesis during subsequent incubation, which resulted in a higher developmental rate to the blastocyst stage. In this experiment, we also observed a greater amount of cell‐free mitochondrial DNA in the culture medium than in those without resveratrol treatment, which suggests that resveratrol may enhance mitochondrial removal in embryos.^66^ Based on these studies, the beneficial effects of resveratrol on vitrified‐warmed oocytes and embryos could be attributed to that activation of mitochondrial biogenesis and degradation by it.

## 6 THE EFFECT OF AGING ON MITOCHONDRIAL KINETICS FOLLOWING VITRIFICATION

**FIGURE 1:**
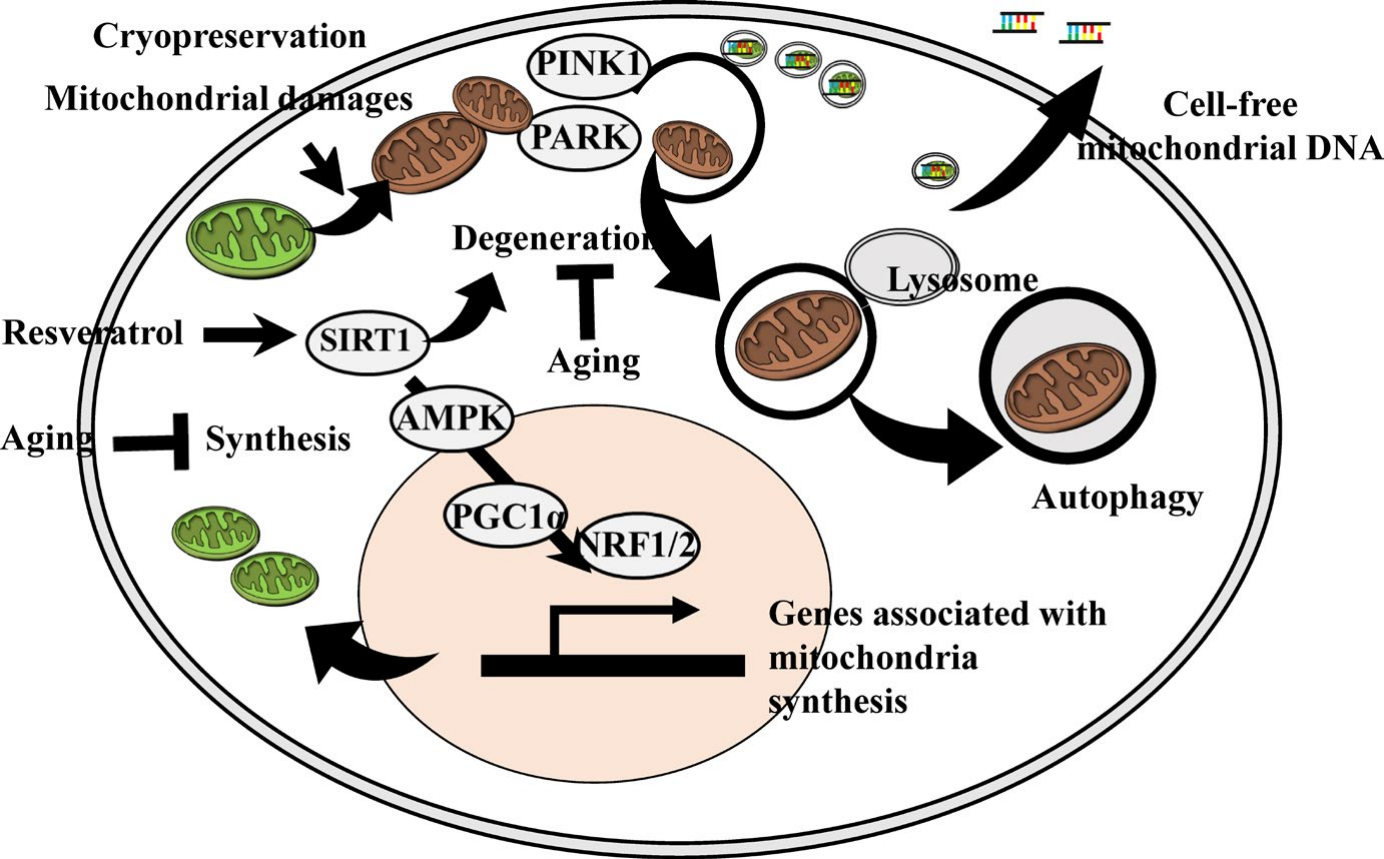
Cryopreservation induces mitochondrial damage. The damaged mitochondria are to be removed from the cells through proteasome and autophagy. The mitochondrial removal likely links to the secretion of cell‐free mitochondrial DNA. Upon resveratrol treatment, upregulation of SIRT1 expression levels further regulates mitochondrial degeneration and biogenesis. Additionally, aging affects the activity of mitochondrial biogenesis and degradation [Colour figure can be viewed at wileyonlinelibrary.com]

Mitochondrial dysfunction and impairment of the ubiquitin proteasome system are major factors in a plethora of aging‐associated diseases,^67^ and aging‐associated low autophagy and mitochondrial biogenesis are causal factors for mitochondrial dysfunction.^68^, ^69^, ^70^, ^71^, ^72^, ^73^ Although a numbers of studies have reported low mitochondrial number and high frequency of mitochondrial dysfunction in oocytes derived from aged mice, humans, and cows,^70^, ^74^, ^75^, ^76^ it is unclear how aging affects the extent of cryopreservation‐induced mitochondrial damage in oocytes and embryos and their recovery from the cryo‐injuries. We reported that when bovine oocytes collected from ovaries are treated with a mitochondrial uncoupler (CCCP) for 2 hours, mitochondrial biogenesis and degradation occurred during the subsequent oocyte maturation period; however, the response to CCCP treatment was observed in the oocytes derived from young cows and not in those from aged cows.^77^ Interestingly, the study showed that the extent of upregulation of phosphate AMPK and SIRT1 and increase in mitochondrial biogenesis after CCCP treatment was less in the oocytes derived from aged cows compared with those derived from young cows. In addition, CCCP treatment did not affect the developmental ability to the blastocyst stage in oocytes of young cows, whereas it was extensively reduced in oocytes of aged cows. The age‐associated low‐level response of mitochondrial number to environmental changes was reported in bovine granulosa cells, where mitochondrial DNA copy number decreased with decreasing oxygen tensions in granulosa cells of young cows but not in those derived from aged cows.^78^ However, a limited number of studies have addressed the effect of aging on the kinetics of mitochondrial number in oocytes and embryos after cryopreservation. We previously showed that the developmental ability of vitrified‐warmed 8‐cell stage embryos to the blastocyst stage did not differ between young cows and aged cows.^79^ Notably, this study showed that mitochondrial DNA copy number in embryos decreased during their development to the blastocyst stage concomitant with an increase in the cell‐free mitochondrial DNA content in the corresponding medium; however, mitochondrial DNA copy number did not decrease in embryos of aged cows, and the amount of the cell‐free mitochondria DNA in the corresponding medium did not increase. This result indicates that the response of mitochondria after vitrification‐induced damage was low in embryos of aged cows; therefore, it is suggested that mitochondrial quality control in response to mitochondrial damage is degraded in aged animals (Figure 1). These findings raise questions because the additive adverse effects of vitrification and aging on the regulation of mitochondrial number and functions might aggravate cryopreservation‐induced mitochondrial damage. We have reported that supplementation of maturation medium with resveratrol activates SIRT1 expression and improves the fertilization outcome of both oocytes of young and aged cows; moreover, SIRT1 inhibitor, E527, diminished this effect.^80^ In addition, upregulation of SIRT1 by resveratrol in oocytes derived from early antral follicles of aged cows increased mitochondrial synthesis and improved the quality of the in vitro*‐*grown oocytes.^81^ However, it is yet to be determined whether resveratrol treatment ameliorates cryopreservation‐induced mitochondrial damage in oocytes and embryos derived from aged female.

## 7 LONG‐TERM CONSEQUENCES OF VITRIFICATION

Generally, cryopreservation does not significantly increase congenital malformation rate; however, it is unclear whether it has any long‐term consequences on the offspring. For example, weight of babies derived from frozen embryos has been reported to be greater than fresh embryo‐derived counterparts for their gestation period.^82^, ^83^, ^84^, ^85^ In a rabbit model, vitrification of embryos increased the birth body and liver wight of offsprings.^86^ In addition, vitrification affected gene expression in sheep embryos,^87^ and variation in methylation pattern of H19/Igf2 was reported.^88^ In another study, Garcia‐Dominguez et al reported that birth weight of rabbit offspring born from vitrified embryos was greater, but the animal had significant low body weight in its adulthood.^89^ Furthermore, the authors reported long‐term transgenerational effects of rabbit embryo vitrification, where not only F1 but also F3 offspring derived from vitrified embryos had differential gene expression profiles.^90^ Causal factors affecting vitrification‐induced changes remain unclear; however, we speculate that the extent of vitrification‐induced damage and the period when the damage occurs in the embryo might affect the phenotype and epigenetic malformation. Therefore, it is important to establish strategies to potentially mitigate the adverse effects of vitrification. Despite the stimulatory effect of resveratrol on the mitochondrial biogenesis and degradation, it is a potent activator of the Sirtuin family, which is potent deacetylase; accumulating evidence has suggested that resveratrol decreases the levels of H3K9 acetylation and increases methylation levels of pronuclei in porcine zygotes.^91^ Furthermore, vitrification of mouse oocytes increased acetylation levels of H3K9 and decreased the amount of methyl cytosine in oocytes, whereas resveratrol treatment ameliorated these changes.^92^ However, it is unclear whether the treatment of embryos with resveratrol induces epigenetic modification or ameliorates vitrification‐induced epigenetic modification.

## 8 CONCLUSION AND FUTURE PERSPECTIVE

Resveratrol has been extensively studied. Till date, 13 859 studies have focused on public medicine using resveratrol as a keyword. Despite the different clinical effects that have been studied for resveratrol, conclusions are yet to be drawn. This could be attributed to individual's variability in terms of lifestyle, diet, inherent genetic background owing to specific ethnicities, gut microbiota, etc In addition, the evidence that resveratrol has beneficial effects on mitochondria in vitrified‐warmed oocyte and embryo can be leveraged to improve their qualities. However, species‐specific differences and donor conditions, such as obesity and aging, have not yet been investigated with respect to resveratrol. Overall, resveratrol can be a useful component because of its prevalence in natural food items, beverages, and supplements.

## DISCLOSURES

*Conflicts of interest*: The author declares no conflicts of interest. *Human rights statements and informed consent*: This article does not contain any studies on human or animal subjects.

## CLINICAL TRIALS REGISTRATION

This study does not include clinical trials.
